# Laparoscopic radical cystectomy with novel orthotopic neobladder with bilateral isoperistaltic afferent limbs: initial experience

**DOI:** 10.1590/S1677-5538.IBJU.2016.0080

**Published:** 2017

**Authors:** Nian-Zeng Xing, Ning Kang, Li-Mming Song, Yi-Nong Niu, Ming-Shuai Wang, Jun-Hui Zhang

**Affiliations:** 1 Department of Urology Beijing Chao Yang Hospital, Affiliate of Capital Medical University, Beijing, Republic of China

**Keywords:** Urinary Bladder Neoplasms, Cystectomy, Laparoscopy, surgery [Subheading]

## Abstract

**Purpose:**

To introduce a new method of constructing an orthotopic ileal neobladder with bilateral isoperistaltic afferent limbs, and to describe its clinical outcomes.

**Materials and Methods:**

From January 2012 to December 2013, 16 patients underwent a new method of orthotopic ileal neobladder after laparoscopic radical cystectomy for bladder cancer. To construct the neobladder, an ileal segment 60cm long was isolated approximately 25cm proximally to the ileocecum. The proximal 20cm of the ileal segment was divided into two parts for bilateral isoperistaltic afferent limbs. The proximal 10cm of the ileal segment was moved to the distal end of the ileal segment for the right isoperistaltic afferent limb, and the remaining proximal 10cm ileal segment was reserved for the left isoperistaltic afferent limb. The remaining length of the 40cm ileal segment was detubularized along its antimesenteric border to form a reservoir. The neobladder was sutured to achieve a spherical configuration.

**Results:**

All procedures were carried out successfully. The mean operative time was 330 min, mean blood loss was 328mL, and mean hospital stay was 12.5 days. The mean neobladder capacity 6 and 12 months after surgery was 300mL and 401mL, respectively. With a mean follow-up of 22.8 months, all patients achieved daytime continence and 15 achieved nighttime continence. The mean peak urinary flow rate was 11.9mL/s and 12.8mL/s at 6 and 12 months postoperatively, respectively.

**Conclusions:**

This novel procedure is feasible, safe, simple to perform, and provides encouraging functional outcomes. However, comparative studies with long-term follow-up are required to prove its superiority.

## INTRODUCTION

Radical cystectomy (RC) with pelvic lymph node dissection (PLND) is the most effective treatment for patients with organ-confined, muscle-invasive, or recurrent high-grade bladder cance (1). Recent advances in the development of laparoscopic instruments, improvements in surgical techniques, and improved surgical confidence and skills now allow the application of laparoscopy to diseases of the pelvic organs, including malignant disease and cases requiring complex reconstruction (2, 3).

Laparoscopic RC (LRC) using a variety of urinary diversion methods has been shown to be feasible and safe, and to be associated with many intraoperative and postoperative advantages (4-6). Various options are available for urinary diversion, among which the orthotopic neobladder has several advantages, including near-normal voiding mechanics, elimination of the need for external appliances, preservation of body image, and superior quality of life (7).

Preservation of renal function is of paramount importance following urinary diversion. It is therefore important to reconstruct the orthotopic low-pressure neobladder so as to reduce long-term impairment of renal function. Here we report on our surgical techniques and preliminary results in 16 patients using LRC with a novel orthotopic ileal neobladder with bilateral isoperistaltic afferent limbs.

## MATERIALS AND METHODS

### Patient selection and preparation

Between January 2012 and December 2013, a total of 16 consecutive patients (15 males and 1 female) underwent LRC with a new orthotopic ileal neobladder with bilateral isoperistaltic afferent limbs in our institution.

The indications and exclusion criteria for LRC and orthotopic neobladder followed the 2012 guidelines of the Chinese Urological Association (8). Indications included: muscle-invasive bladder cancer stage T2–4a, N0–Nx, M0

- high-risk and recurrent non-muscle-invasive tumors
- bladder cancer stage T1G3
- extensive non-muscle-invasive disease that could not be controlled by transurethral resection and intravesical therapy.

### Exclusion criteria included

- patient refusal of LRC
- the presence of contraindications to LRC, including distant metastasis; an American Society of Anesthesiologists (ASA) score >3; severe cardiac insufficiency; and decompensated pulmonary function that made the patient unable to tolerate pneumoperitoneum
- the presence of contraindications to neobladder, including tumor in the urethra, urethral stricture, abnormal abdominal straining, and decompensated renal function.

Out of the total of 16 patients, 8 presented with a primary bladder tumor and 8 with recurrent disease. All had had previous transurethral resection of the bladder tumor and intravesical chemotherapy. In our female case, the radical cystectomy included removal of the bladder, uterus, and distal ureters (9). The patient had pelvic lymphadenopathy indicated through CT preoperatively; we performed the extended PLND, and the pathological result was negative. All patients were diagnosed by cystoscopy and tumor biopsy. No distant metastasis was identified in any patient. Patients were evaluated preoperatively with cystoscopy, tumor biopsy, intravenous urography, ultrasonography, bone scan, computed tomography (CT), and/or magnetic resonance imaging (MRI).

All patients were determined by cystoscopy to be free of tumor in the urethra. Demographic and clinical parameters including age, sex, body mass index (BMI), ASA score, and clinical stage were assessed. Perioperative data were analyzed, including operative time, estimated blood loss, tumor size and histopathology, along with the incidence of complications.

All patients underwent bowel preparation by oral self-administration of 4L polyethylene glycol with electrolytes the day before the surgical procedure. On the morning of surgery, broad-spectrum intravenous antibiotics were administered. Compression stockings were applied to the lower extremities before induction of anesthesia in order to prevent deep vein thrombosis.

### Surgical technique

Laparoscopic radical cystoprostatectomy or radical cystectomy was performed in all male and 1 female patients, respectively, followed in all patients by pelvic lymphadenectomy.

### Patient positioning and port placement

General anesthesia was administered by tracheal intubation. The patient was placed in a supine steep Trendelenburg position and a nasogastric tube was inserted. A 16Fr Foley catheter was positioned, and 50mg epirubicin was perfused immediately for intravesical chemotherapy before the operation.

Pneumoperitoneum was obtained with a Veress needle. A primary 10mm laparoscopic trocar was placed at the level of the umbilicus. After inspection of the abdominal cavity, 4 other trocars were placed in a fan-shape. Two 12mm trocars were placed in ports 2cm below the umbilicus on the mid-clavicular line on both sides, and two 5mm trocars were placed 2–3cm superior and medial to the anterior superior iliac spines on each side.

### Laparoscopic radical cystoprostatectomy

Our procedure comprised the following10 steps:

1. The adhesion between the sigmoid colon and the pelvis was dissociated, and the left ureter was mobilized.
2. The right ureter was mobilized.
3. A transverse peritoneotomy was made in the Pouch of Douglas.
4. The ampullae of the vas deferens were transected bilaterally, and the seminal vesicles were dissected and maintained en bloc with the bladder. Denonvillier’s fascia was incised, and Denonvillier’s space between the rectum and the prostate was developed as far as the apex of the prostate.
5. The anterior bladder wall and the space of Retzius were exposed.
6. The endopelvic fascia were incised bilaterally.
7. The puboprostatic ligament was dissected, and the dorsal vein complex was suture-ligated.
8. The lateral pedicles of the bladder and the prostate were bilaterally divided with LigaSure® (Covidien, Dublin, Ireland) if the neurovascular bundle did not need to be preserved. Otherwise, the bladder and prostate lateral pedicles were divided with Hem-o-lok® (Teleflex Medical, Research Triangle Park, NC, USA), clips and scissors.
9. The prostatic capsule was incised from 0.5–1.0cm to the apex of the prostate, the urethra was dissociated, the catheter removed, and the proximal urethra was clipped with Hem-o-lok® to prevent leakage of urine. For absolute certainty, the prostatic capsule was reserved about 0.5–1.0cm from the apex of the prostate and the prostate and urethra were removed.
10. Both ureters were clipped and divided close to the bladder, and the distal ureteral margin was sent for frozen section evaluation. The proximal cut end of each ureter was temporarily occluded with clips to facilitate hydrostatic distension.

### Bilateral pelvic lymph node dissection

After RC was accomplished, PLND was performed, beginning with right lymphadenectomy. The criteria of a standard PLND were carried out, namely bifurcation of the common iliac artery proximally, the genitofemoral nerve laterally, the circumflex iliac vein and lymph node of Cloquet distally, and the hypogastric vessels posteriorly, including the obturator fossa. The removal of the lymph nodes was performed using a small endobag. Extended PLND was performed if there were positive results from frozen lymph node evaluation or if CT or MRI indicated pelvic lymphadenopathy.

### Orthotopic ileal neobladder with bilateral isoperistaltic afferent limbs

Through the 8cm midline incision, the ileum was extracted from the peritoneal cavity, manual end-to-end anastomosis was performed to restore the continuity of the ileum, and the mesenteric window was closed to prevent internal hernia.

**Figure 1:**
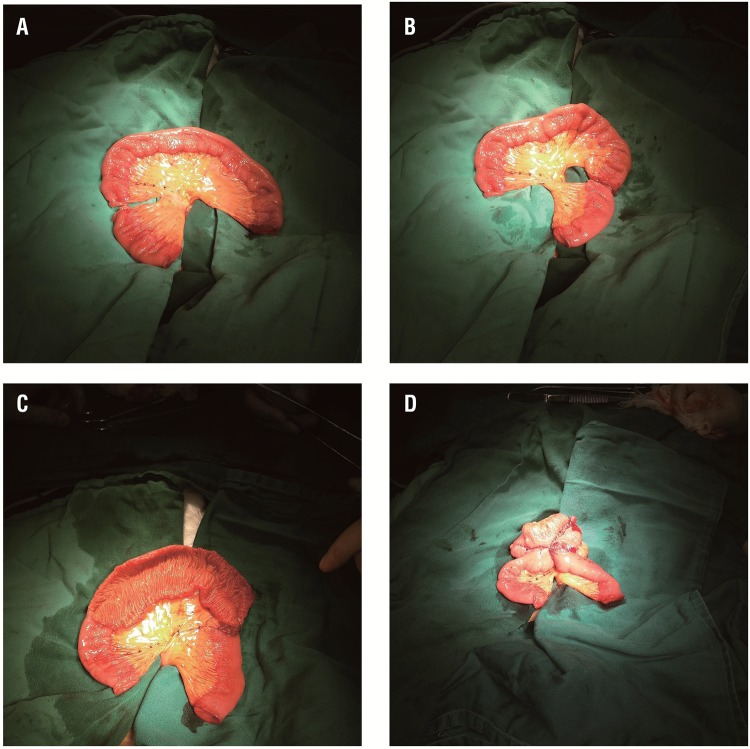
– A) The orthotopic ileal neobladder with bilateral isoperistaltic afferent limbs. The proximal 20 cm of the ileum was divided into 2 segments for the bilateral isoperistaltic afferent limbs. B) The proximal 10 cm ileal segment was moved to the right side. C) The remaining 40 cm long segment was cleaned and detubularized along its antimesenteric border. D) The anterior wall of the neobladder was folded forward and the free edges were sutured to achieve a spherical configuration.

To construct the neobladder, an ileal segment 60cm long was isolated 25cm proximal to the ileocecum. The proximal 20cm of the ileal segment was divided into 2 parts for bilateral isoperistaltic afferent limbs. The proximal 10cm of the ileal segment was moved to the distal end of the ileal segment for the right isoperistaltic afferent limb, and the remaining proximal 10cm ileal segment was reserved for the left isoperistaltic afferent limb. Then, the remaining length of the 40cm ileal segment was detubularized along its antimesenteric border. The posterior wall of the neobladder was closed by continuous suturing of adjacent detubularized ileal walls using 2-0 Vicryl suture. The anterior wall of the neobladder was folded forward and the free edges were sutured to achieve a spherical configuration (Figure-1).

Before completing the suture of the neobladder, bilateral 6Fr ureteral single-J stents were delivered into the bilateral renal pelvis through the urethra, neobladder, limbs and ureters. The ureters were spatulated for about 1.5cm and the ureters were end-to-end anastomosed with the ipsilateral limb in a continuous manner using two separate 3-0 Vicryl sutures for each ureter. Sutures were used for the posterior and anterior walls. In this procedure, unlike in other types of urinary diversion, none of the ureter needed to be tunneled under the mesosigmoid to the contralateral side.

The neobladder was brought into the pelvis, and the urethra-ileal anastomosis was completed using a running suture intracorporeally. A 22Fr Foley catheter was then positioned.

### Postoperative care

The reservoir was irrigated with 1.6% sodium bicarbonate solution and gentamicin saline with aspiration of the mucus through the Foley catheter every 4h starting on postoperative day 3. The tubal drains were removed after fluid drainage had ceased. The ureteral stents were removed 11–14 days after surgery. The urethral catheter was maintained for 15 days, a pouchography was performed, and the urethral catheter was removed if there was no extravasation.

Patients were instructed to void while sitting after removal of the urethral catheter to facilitate good pouch evacuation. The voiding interval was gradually increased from 2 to 4h. The goal was a final bladder capacity of 400–500mL with urinary continence after 12 months.

### Follow-up

Postoperative follow-up was conducted at 3-month intervals during the first year, at 6-month intervals during the second year, and annually thereafter.

Follow-up visits consisted of a history, physical examination, and routine biochemical profile. Ultrasonography of the abdomen, urography and chest x-rays were performed at 3, 6 and 12 months postoperatively, then annually unless otherwise clinically indicated. Abdominal/pelvic CT scans were performed 6 months postoperatively and annually thereafter.

Radiographic studies to evaluate the upper urinary tract included excretory urography, CT and renal ultrasonography. Serum determination of blood urea nitrogen and creatinine along with routine chemistry studies were performed at each follow-up visit.

The estimated glomerular filtration rate (eGFR) was calculated preoperatively and at various intervals after surgery. It was calculated with an equation developed by adaptation of the Modification of Diet in Renal Disease (MDRD) equation on the basis of data from Chinese chronic kidney disease patients (10).

Continence status and voiding pattern were determined by telephone interview. Continence was strictly defined as good if the patient was completely dry without the need for any protection, satisfactory if no more than 1 pad was required during the day or night, and unsatisfactory if the patient was using more than 1 pad during the day or night. The voiding pattern was classified as: being able to void to completion without the need for catheterization; requiring any form of intermittent catheterization for residual urine; or being unable to void and requiring continuous intermittent catheterization.

## RESULTS

Patient demographic and perioperative data are presented in Table-1. There were 15 men and 1 woman with a mean age of 65 years (range: 54–77 years) and a mean BMI of 25.0 (range: 21.3–28.1). The mean ASA score was 2.3. All procedures were carried out successfully without conversion to open surgery. The mean operative time was 330 min (range: 260–410 min). The mean estimated blood loss was 328mL (range: 200–600mL), and 1 (6%) patient required transfusion.

**Table 1:** Demographics and surgical outcomes.

Age mean±SD (range), year	64.8±6.0 (54-77)
ASA mean±SD (range)	2.3±0.4(2-3)
Male, n (%)	15（(93)）
BMI mean±SD (range), kg/m^2^	25.0±1.8 (21.3-28.1)
Operative time mean±SD (range),min	330±47(260-410)
Estimated blood loss mean±SD (range), mL	328±113 (200-600)
Blood transfusion n (%)	1 (6)
Oral fluids intake time mean±SD (range), day	4.9±0.8 (4-6)
Drainage tube remove mean±SD (range), day	8.1±1.5 (6-11)
Hospital stay, mean±SD (range), day	12.5±1.6(10-16)
**Complications,n**	
**Intraoperative complications**	
Obturator nerve injured	1
**Early complications (**≤**30 days)**	
Urine leakage	1
Urinary infection	1
**Late complications**（**(≤90 days)**	
Adhesive intestinal obstruction	1
**Pathology results**	
Transitional cell carcinoma n. (%)	15(94%)
Adenocarcinoma n. (%)	1(6%)
Number of pelvic lymph nodes	16.1±3.3 (8-22)
Patient with positive pelvic lymph node (%)	1(6%)
Follow-up median (range), month	26(16-39)

**BMI** = Body Mass Index; **ASA** = American Society of Anesthesiologists; **SD** = standard deviation.

Patients were ambulatory on postoperative day 1 or 2, and the mean time to resumption of oral intake was 4.9 days (range: 4–6 days). The drainage tube was removed at a mean of postoperative day 8.1 (range: 6–11 days), the bilateral ureteral stent was removed on postoperative day 14, and the Foley catheter was removed on postoperative day 14 or later. The mean hospital stay was 12.5 days (range: 10–16 days).

### Complications

Complications were graded according to the modified Clavien classification system (11). In this series, we observed 6% (1/16), 13% (2/16) and 6% (1/16) intraoperative, early (≤30 days) and late complications (≤90 days), respectively (Table-1). All early and late complications were minor complications (grade I–II). Complications were mostly related to the orthotopic neobladder urinary diversion. The obturator nerve was injured in one case, but laparoscopic nerve anastomosis was carried out successfully and the patient recovered without any dysfunction.

### Neobladder function

At 12 months postoperatively, the daytime and nighttime continence rates of the 15 male patients were 100% (15/15) and 93% (14/15), respectively. One patient used small/mini pad at night. The pouch capacity and the residual volume were measured by ultrasonography. The mean maximal pouch capacity was 297mL (range: 200–410mL) and 400mL (range: 345–480mL) at 6 and 12 months postoperatively, respectively. The mean residual volume was 16.6mL (range: 0–40mL) and 28mL (range: 0–60mL) at 6 and 12 months postoperatively, respectively. The mean peak flow rate was 11.5mL/s (range: 6.5–16.5mL/s) and 12.2mL/s (range: 8.8–17.2mL/s) at 6 and 12 months postoperatively, respectively.

The daytime and nighttime continence of the female patient was satisfied. The maximal pouch capacity was 350mL and 410mL at 6 and 12 months postoperatively, respectively. The residual volume was 20mL and 50mL at 6 and 12 months postoperatively, respectively. The peak flow rate was 20mL/s and 20.5mL/s at 6 and 12 months postoperatively, respectively (Table-2).

**Table 2:** eGFR, Urinary continence and neobladder function of 16 patients at postoperative 12 months.

eGFR (range), mL per minute/1.73 m^**2**^	
preoperatively	72.1±12.4(48.4-89.6)
postoperatively 12months	69.1±12.0(40.9-85.5)
**Continence**	
Day continence, n	16
Night continence, n	15
Satisfactory continence, n (%)	15(94%)
Total incontinence, n (%)	1(6%)
**Neobladder functions**	
Maximal pouch capacity mean (range), mL	401 (345-480)
Residual volume mean(range), mL	30 (0-60)
Peak flow rate mean(range), mL/s	12.8 (8.8-20.5)

### Pathology data

The pathology results are presented in Table-1. In this study, 15/16 (94%) patients had transitional cell carcinoma, and 1/16 (6%) patient had adenocarcinoma. The mean number of lymph nodes removed was 16.1 (range: 8–22). Five patients who had high-grade transitional cell carcinoma and 1 patient with positive lymph nodes received adjuvant chemotherapy.

### Follow-up and survival data

**Figure 2:**
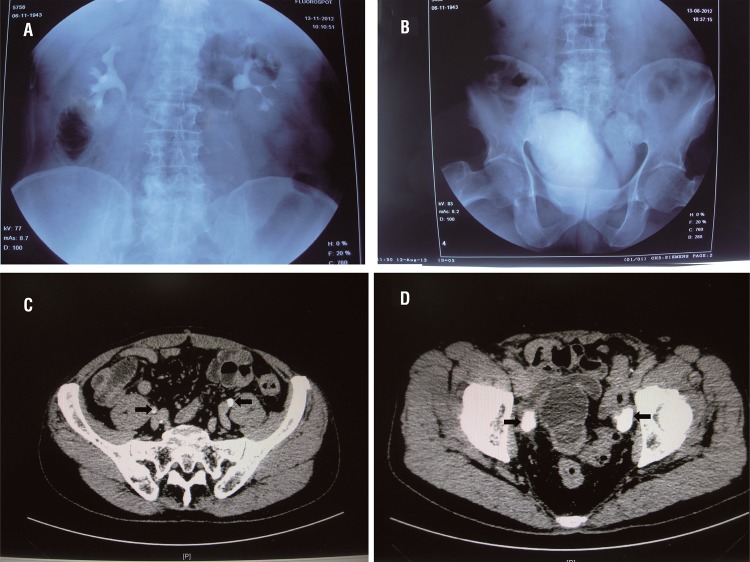
Image examinations from follow-up. A) An IVP showed the bilateral pelvis and ureters were normal and there were no appearance of hydronephrosis (POD 10 months). B) An IVP showed the shape of the neobladder. C) Delay phase of a enhanced CT scan showed there were no appearance of the dilation of the bilateral ureters (black arrows). D) Delay phase of the enhanced CT scan showed the appearance of the bilateral isoperistaltic afferent limbs (black arrows) and the neobladder.

After a median follow-up of 22.8 months (range: 16–36 months), 12 patients were alive with no evidence of local recurrence or distant metastasis. Four patients were diagnosed as lung metastases and received adjuvant chemotherapy. One patient died 18 months postoperatively. The median eGFR was 72.1mL per minute/1.73m2 preoperatively and 69.1mL per minute/1.73m2, 1 year postoperatively. There was no deterioration in renal function in any of the patients during follow-up. The intravenous pyelogram and enhanced CT scan showed that no hydronephrosis had occurred (Figure-2).

## DISCUSSION

The advances that have been made in both the design of instruments and laparoscopic techniques have stimulated the development of laparoscopic surgery. Nowadays, LRC is considered to be a safe, feasible and minimally invasive alternative to RC with fewer overall complications, less blood loss, shorter length of hospital stay, shorter time to regular diet, and reliable pathologic and oncologic efficacy (12).

We reported our first laparoscopic radical cystectomy and urinary diversion in 2005, and since then more than 600LRCs have been performed by experienced surgeon Nianzeng Xing. Most of the neobladder reconstructions were performed extracorporeally through a 5–8cm incision.

Hautmann et al. initially reported an orthotopic low-pressure reservoir using detubularized ileum in 1988 (13). One year later, Studer et al. described a new technique using a long, afferent and isoperistaltic ileal segment for the construction of a neobladder with good functional results (14). In many institutional centers worldwide, orthotopic neobladder has now replaced the ileal conduit as the standard form of reconstruction.

Preservation of upper tract integrity and function is one of the essential requirements when a urinary bladder substitute is indicated. Following Hinman’s principles (15), there is general agreement that detubularization of the bowel is required to nullify the pressure waves created by peristalsis and to obtain maximum capacity from a given length of bowel. Studer and coworkers proposed the use of an isoperistaltic long afferent loop for reflux prevention. They maintained that the unidirectional peristalsis of the ureters and the afferent tubular ileal segment sufficiently protected the upper urinary tracts following ileal bladder substitution up to a decade after urinary diversion (16).

However, the Studer pouch has only one afferent limb, which is anastomosed with bilateral ureters. The left ureter has to be tunneled under the mesosigmoid to the right side in order to be implanted in the afferent ileal part. In 2013, Studer et al. retrospectively evaluated the records of 74 patients treated for unilateral or bilateral nonmalignant ureteroileal strictures, indicating that left ureteroileal strictures were almost twice as common as right strictures, and, compared with right strictures, they were significantly more often larger than 1cm (17). They concluded that the additional mobilization of the left ureter as it was brought to the right side could worsen the blood supply and thus lead to the development of ureteroileal stricture as a result of chronic ischemia.

In our study, the ureters were anastomosed with the ipsilateral limb of the neobladder without excessive mobilization. No patients developed an ureteroileal stricture during the 2-year follow-up, and this advantage should certainly be demonstrated by long-term follow-up. The rate of ureteroileal stricture was reduced because:

- there is no need for excessive mobilization and devascularization of the left ureter
- none of the ureter needs to be tunneled under the mesosigmoid to the contralateral side
- the diameter of the anastomosis is wide enough.

Stein and Skinner reported on the orthotopic T-pouch ileal neobladder incorporating a serosa-lined ileal antireflux technique and concluded that it is an extremely effective and versatile flap-valve method that can be applied to the construction of continent urinary diversions (18). However, it is a complex surgical procedure and, in addition, the ureter has to be transposed. We have performed the T-pouch procedure since 2009. It is a good orthotopic neobladder with regard to offering good kidney protection and urinary continence, but it is too complicated, especially for intracorporeal construction.

Y-shaped orthotopic neobladder was reported by Fontana et al. (19). The procedure of Y-shaped neobladder construction was described as follows: the isolated intestinal segment was arranged in a Y-shape with 2 central segments of 14cm and two limbs of 6cm. The 2 central segments were brought together and detubularized. The ureters were directly anastomosed to the open ends of the limbs of the neobladder. The Y-shaped neobladder is quite different from our neobladder. One important difference is our pouch is a true globularized neobladder with increased capacity and decreased pressures. Furthermore, our pouch has bilateral isoperistaltic afferent limbs, whereas although the Y-shaped neobladder has bilateral afferent limbs, only one of the limbs is isoperistaltic.

In 2002, Gill et al. first reported their initial clinical experience with LRC with intracorporeal orthotopic ileal neobladder in 2 patients (20). Since then, the robotic-assisted approach is most commonly used in intracorporeal neobladder construction.

In 2015, Shao et al. presented their experience of LRC with intracorporeal orthotopic ileal neobladder in 50 cases (21). However, it still does not use a special neobladder construction for intracorporeal approach. Our novel orthotopic neobladder can be carried out purely laparoscopically. We began to perform LRC and intracorporeal neobladder construction with this neobladder last year. The results are encouraging, and will be reported later.

Monitoring both eGFR and postoperative hydronephrosis permitted early detection of urinary obstruction. Prompt surgical treatment prevented renal deterioration in this subgroup of patients. In our study both the eGFR and imaging showed the result were satisfied.

The main limitation of this study is the relatively small sample size, short follow-up time, and lack of control group. However, the aim of this study was to report our experience with constructing the novel neobladder as performed by one surgical team. A larger, multicenter, comparative study with long-term follow-up is needed to confirm its superiority.

This novel procedure is feasible and safe to perform with encouraging functional outcomes. It does not require removal of the left ureter to the right side to anastomose with the reservoir, and the ureters are sutured to the afferent limbs end-to-end. The bilateral isoperistaltic afferent limbs of the neobladder protect the morphology and function of the upper urinary tract.

### Ethical Standards

The study received approval from the research ethics board of Chaoyang Hospital.

## ARTICLE INFO

Int Braz J Urol. 2017; 43: 57-66
